# Supplementary figures and images for: Novel small RNAs expressed by Bartonella bacilliformis under multiple conditions reveal potential mechanisms for persistence in the sand fly vector and human host
Source: PLoS Negl Trop Dis. 2020 Nov 20;14(11):e0008671. doi: 10.1371/journal.pntd.0008671 (PMC7717549; doi:10.1371/journal.pntd.0008671)

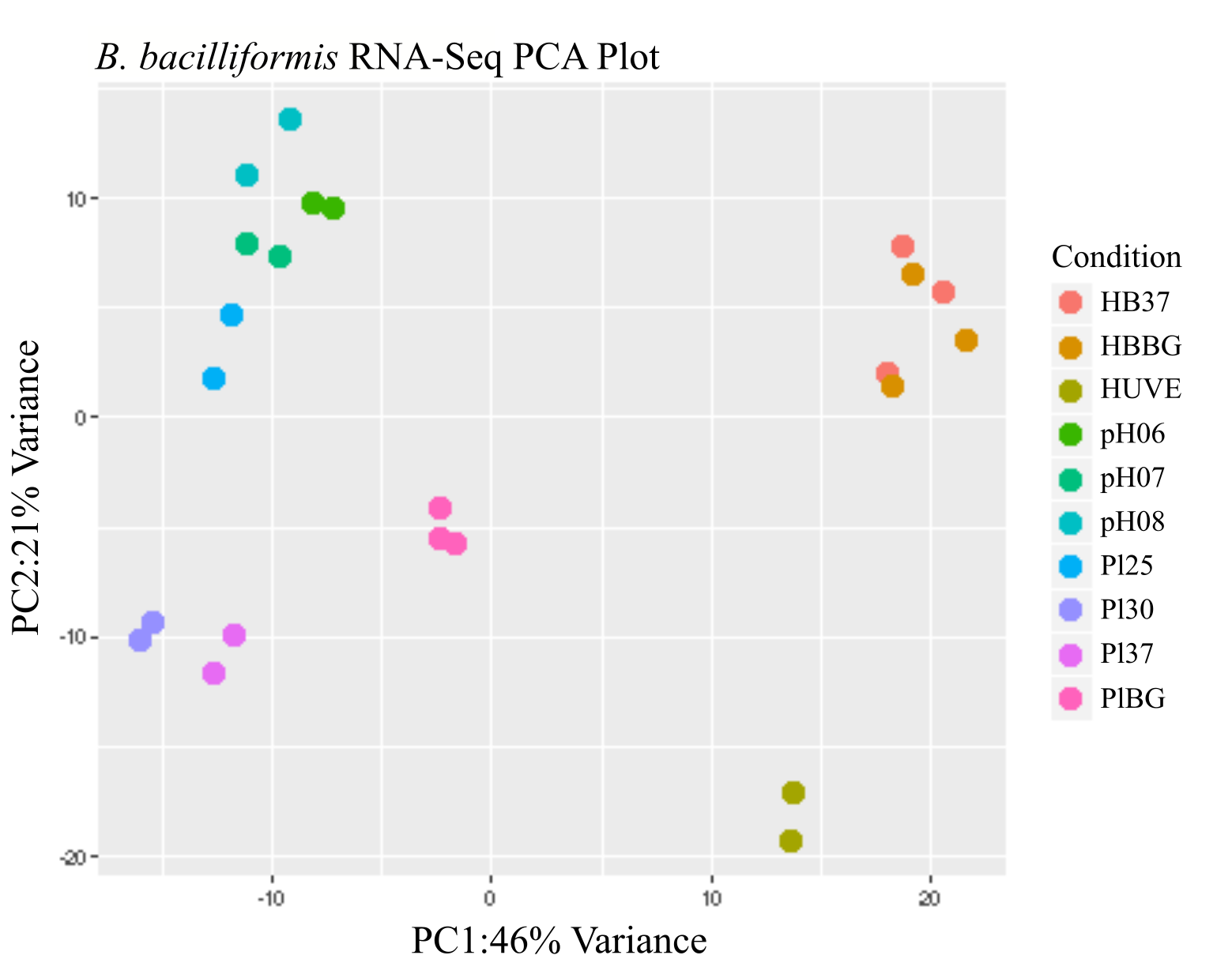

Supplement: S1 Fig — Axes indicate the percentage of total variance that can be accounted for by two principle components. Colored dots indicate the retained biological replicates of the RNA-Seq analyses, and their distance apart is representative of overall relatedness in gene expression profiles. Experimental conditions are shown on the right. (TIFF) [file pntd.0008671.s001.tiff]

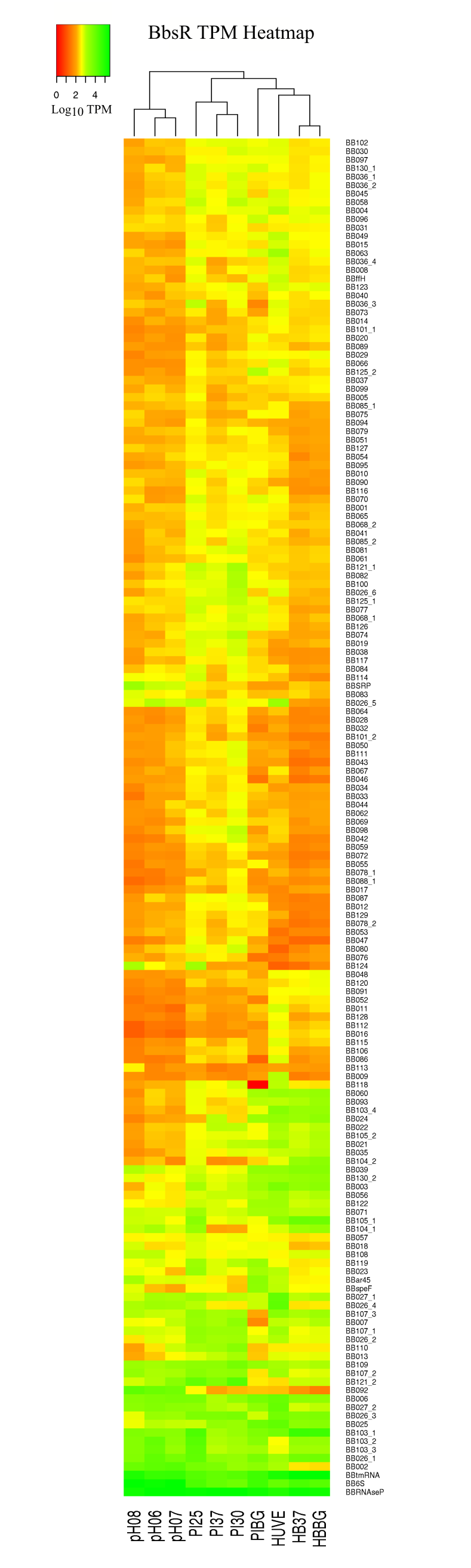

Supplement: S2 Fig — Heatmap of B. bacilliformis sRNA TPMs across the tested conditions (shown at the bottom). sRNAs group vertically based on similarity in expression patterns. Conditions group horizontally based on similarity in overall expression patterns. The log10 of the TPM value for each sRNA is indicated by a color gradient. (TIFF) [file pntd.0008671.s002.tiff]

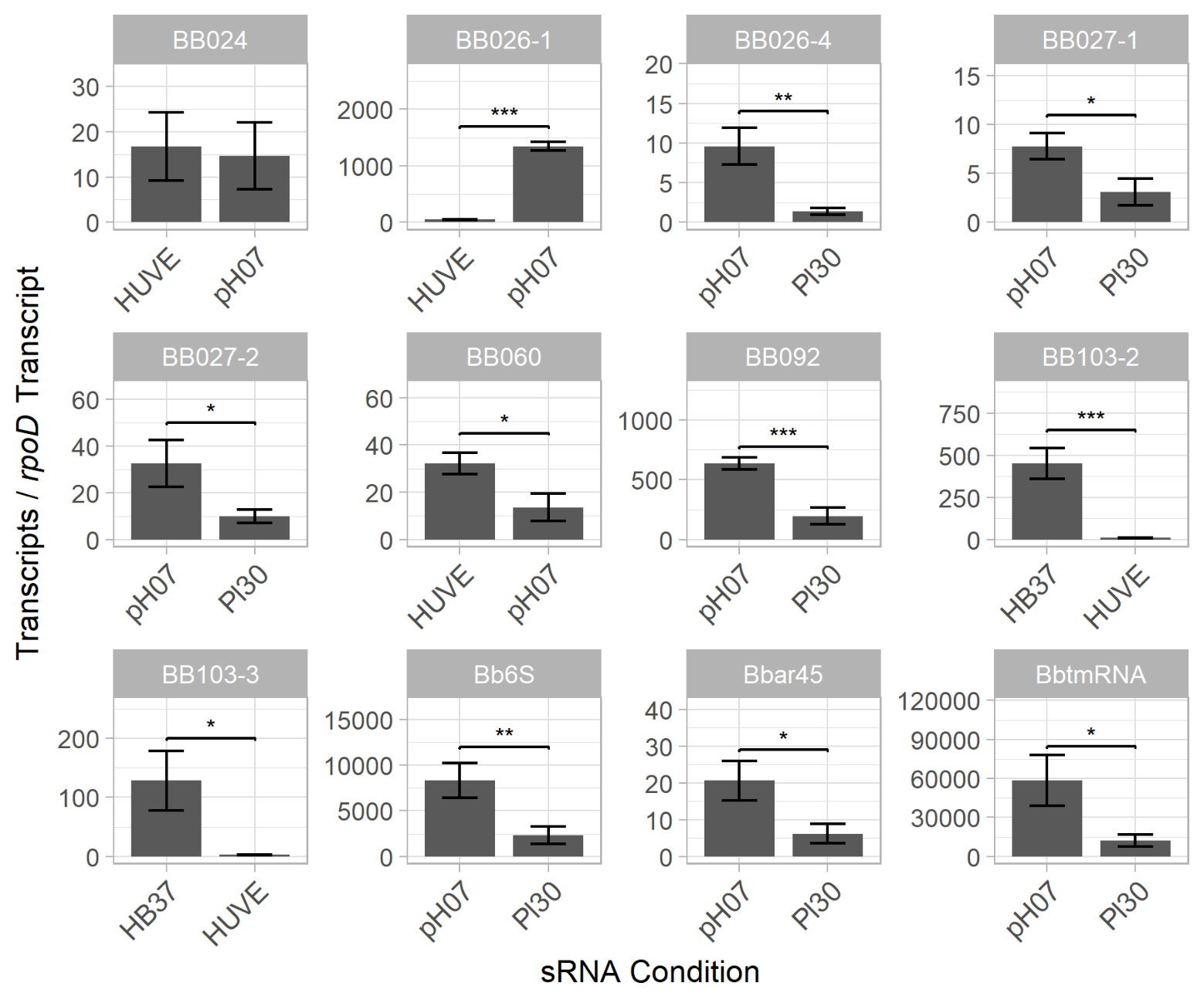

Supplement: S3 Fig — Faceted bar graph displaying the number of sRNA transcripts / rpoD transcript for select, differentially-expressed sRNAs and BB024, which was not shown to be differentially expressed. The condition / source of the total RNA is noted on the x-axis. Significance was determined by students t-test (N = 9; * = p<0.05, ** = p<0.01, *** = p<0.001). (TIFF) [file pntd.0008671.s003.tiff]

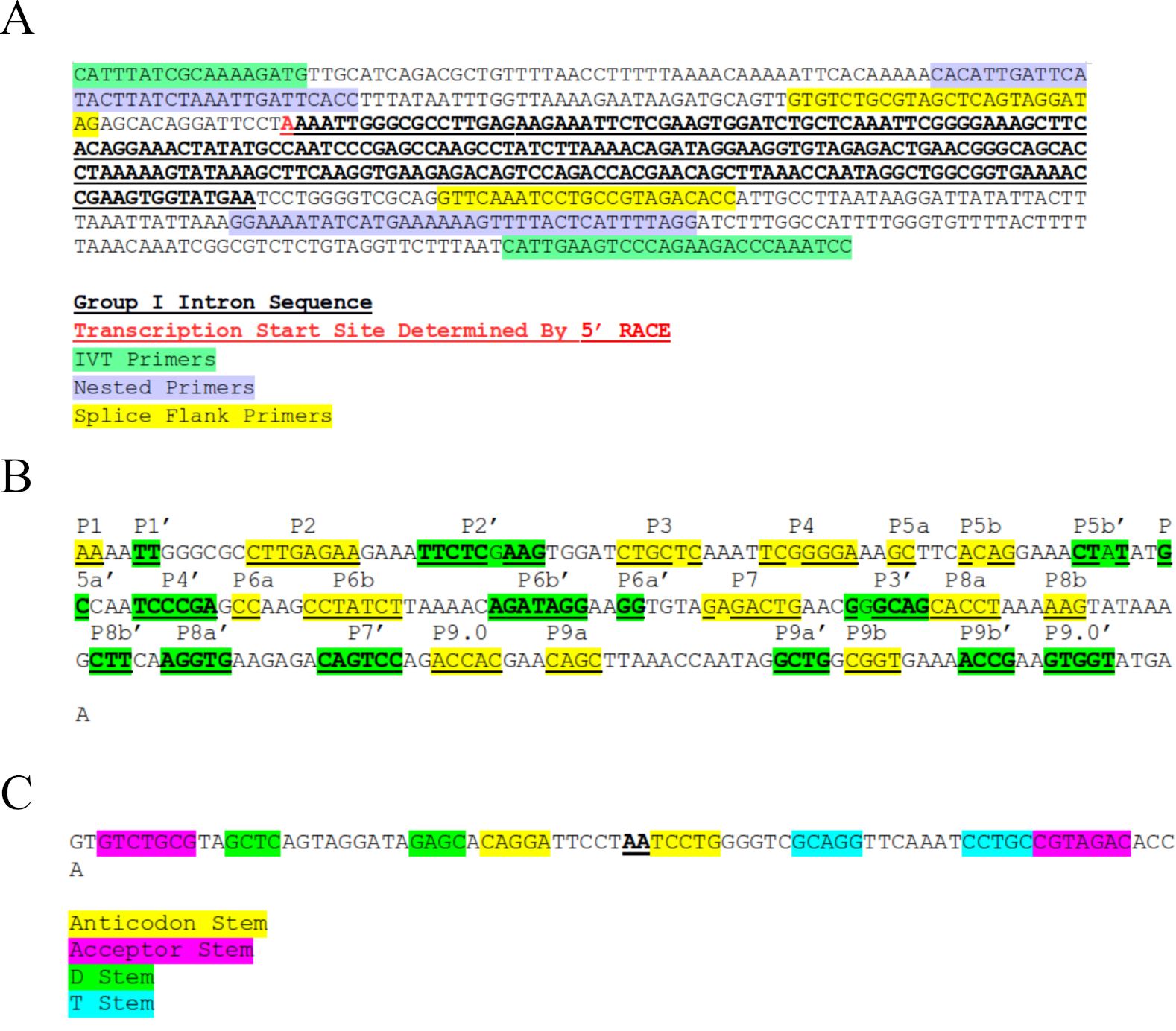

Supplement: S4 Fig — A) Nucleotide sequence of BbgpI (bolded and underlined) and flanking chromosomal regions. Primer binding sites used for in vitro transcription (IVT) and PCR assays designed to show splicing of BbgpI in vitro and in vivo are indicated. B) Sequence of BbgpI outlining the conserved, characteristic stem structures (P1 to P9) with putative base pairings highlighted in green and yellow. Nucleotides predicted to participate in base pairing are bolded and underlined. C) Sequence coding for the tRNACCUArg immediately flanking BbgpI. The two bolded underlined nucleotides represent the ends of the spliced out BbgpI. Conserved tRNA features are also outlined. (TIFF) [file pntd.0008671.s004.tiff]

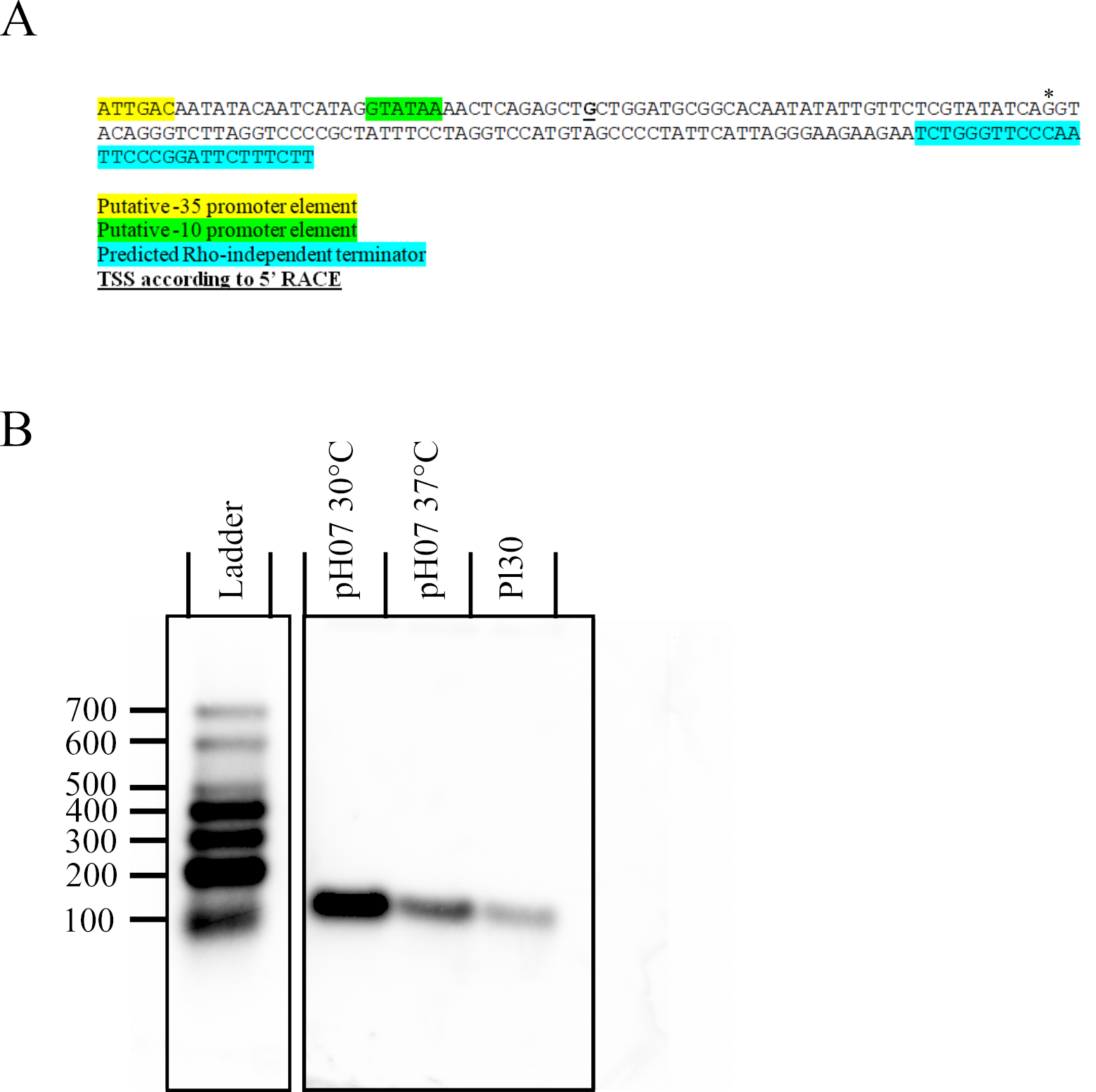

Supplement: S5 Fig — A) Nucleotide sequence of the bbsR9 gene with predicted promoter elements and Rho-independent terminator plus experimentally-determined TSS’s, highlighted in various colors or underlined, respectively. An asterisk indicates the alternative TSS found by 5' RACE analysis. B) Northern blot analysis of BbsR9 expression under the indicated conditions. The RNA ladder (2 min exposure) and resolved total RNA samples (30s exposure) were from the same blot but imaged using different exposure times. (TIFF) [file pntd.0008671.s005.tiff]
